# Central Nervous System Involvement in Common Variable Immunodeficiency: A Case of Acute Unilateral Optic Neuritis in a 26-Year-Old Italian Patient

**DOI:** 10.3389/fneur.2018.01031

**Published:** 2018-11-30

**Authors:** Elena Abati, Irene Faravelli, Francesca Magri, Alessandra Govoni, Daniele Velardo, Delia Gagliardi, Eleonora Mauri, Roberta Brusa, Nereo Bresolin, Giovanna Fabio, Giacomo Pietro Comi, Maria Carrabba, Stefania Corti

**Affiliations:** ^1^Neurology Unit, Department of Pathophysiology and Transplantation (DEPT), Dino Ferrari Centre, Neuroscience Section, Foundation IRCCS Ca' Granda Ospedale Maggiore Policlinico, University of Milan, Milan, Italy; ^2^Department of Internal Medicine, IPINet Primary Immunodeficiency Centre for Adult Care, IRCCS Foundation Ca' Granda Ospedale Maggiore Policlinico, University of Milan, Milan, Italy

**Keywords:** common variable immunodeficiency, optic neuritis, optic neuropathy, clinically isolated syndrome, autoimmunity, primary immunodeficiencies

## Abstract

Common Variable Immunodeficiency (CVID) is a group of heterogeneous primary immunodeficiencies sharing defective B lymphocytes maturation and dysregulated immune response and resulting in impaired immunoglobulin production. Clinical picture encompasses increased susceptibility to infections, hematologic malignancies, inflammatory, and autoimmune diseases. Neurological manifestations are uncommon and optic neuritis has been previously reported only in one case with bilateral involvement. We hereby report a case of a 26-year-old man affected by CVID undergoing regular immunoglobulin supplementation, who presented with acute unilateral demyelinating optic neuritis and lymphocytic pleocytosis in the cerebrospinal fluid. A variety of infectious, inflammatory, and neoplastic conditions were excluded and a diagnosis of clinically isolated optic neuritis was made. The patient was treated with a short course of intravenous steroids with complete recovery. Overall, this case expands our current knowledge about clinical spectrum of complications in CVID and highlights the need for further research about this complex disease.

## Background

Common Variable Immunodeficiency (CVID) is a group of primary immunodeficiencies characterized by an impairment in antibody production related to B cell intrinsic or extrinsic defects (1). CVID is defined by increased susceptibility to infection, autoimmune manifestations, granulomatous disease, and unexplained polyclonal lymphoproliferation associated to markedly reduced levels (2 SD below the mean) of serum immunoglobulin (Ig) G, as well as IgA and/or IgM, failed antibody response to natural infections or vaccine immunization and reduction of switched memory B cells, with the exclusion of secondary causes of hypogammaglobulinemia and no evidence of profound T-cell deficiency or T-cell proliferation (1–3). Clinical manifestations are heterogeneous and include increased susceptibility to infections, autoimmune manifestations, malignancies, granulomatous disease, and unexplained polyclonal lymphoproliferation, affecting multiple organ systems (2, 4–7). Among autoimmune disorders, the most common are thrombocytopenia, hemolytic anemia, and rheumatologic disorders (4, 6–8). Neurologic complications are rare, and include a variety of central nervous system (CNS) infections, the most common being bacterial meningitis and viral meningoencephalitis, autoimmune/inflammatory encephalomyelitis or peripheral neuropathies (9). Only one case of bilateral posterior optic neuritis is reported (10).

Acute optic neuritis (ON) is caused by inflammation of the optic nerve and represents the most common optic neuropathy affecting young adults (11). Typical ON presents with subacute, monocular visual defects, which include a decrease in visual and contrast acuity, focal campimetric deficits, diffuse blurring or fogging of vision, and dyschromatopsia, generally associated with pain on eye movements (11). Relative afferent pupillary defect is also commonly observed (11). In the majority of cases, ON is a manifestation of multiple sclerosis (MS). Other potential causes include Acquaporin 4 (AQP4)-IgG-positive neuromyelitis optica spectrum disorders (NMOSD) (12) and Myelin oligodendrocyte glycoprotein (MOG)-IgG-associated encephalomyelitis (13), in addition to systemic autoimmune disorders such as connective tissue diseases, vasculitides, or sarcoidosis (11). A variety of bacterial, viral, parasitic, and fungal infectious agents can also be responsible for ON (14).

Here, we present a case of a young man with CVID and acute onset of unilateral inflammatory optic neuritis.

## Case presentation

A 27-year-old man was admitted to our Emergency Department for a 3-days-history of blurry vision in his left eye. His medical history included CVID: during early childhood, he experienced multiple respiratory tract infections and selective IgA deficiency was diagnosed. At the age of 11, he developed splenomegaly and multiple abdominal and thoracic lymphadenopathies. At the age of 14, histological analysis of an inguinal lymph node was performed, showing reactive lymphadenopathy and no evidence of lymphoproliferative disease. Serum IgG were 593 mg/dL, IgM 50 mg/dL, IgA 6 mg/dl. Few months later he developed severe immune thrombocytopenic purpura (ITP), successfully treated with a course of intravenous immunoglobulins. Autoimmune/lymphoproliferative syndrome (ALPS) was excluded based on negative molecular analysis for *TNFRSF6*. No further investigations for lymphoproliferation and hypogammaglobulinemia were done at that time. At the age of 21, he experienced recurrent prostatic infections and Herpes Zoster reactivation on the trunk. After a long delay, at the age of 23, diagnosis of CVID was made according to the criteria developed by the European Society for Immune Deficiencies (ESID) and the International Consensus on CVID (2, 3). He presented recurrent sinusitis, splenomegaly, laterocervical, axillary, and inguinal lymphoadenopathy. Total IgG, IgM, and IgA count at diagnosis was 150 mg/dL, 6 mg/dl, and 0, respectively. Lymphocytes immunophenotyping and maturative analysis revealed normal levels of circulating B cells (341/μL), failure of B lymphocytes maturation with reduced levels of switched memory B cells (0.6%), and increase in CD21 low subpopulation (22%). T and NK cell subsets were in normal range. Serum β2-microglulin was 3.54 mg/dL (n.r. 0.8–2.2). Molecular analyses for HIV, EBV, CMV, *Toxoplasma* were negative. Lymphoma was ruled out by bone marrow trephine biopsy and total body Computed Tomography (CT)-scans showed no change in previously described thoracic and abdominal lymphadenopathies and splenomegaly. Other causes of secondary hypogammaglobulinemia were excluded. Therefore, replacement therapy with intravenous immunoglobulins (0.4 g/kg every 3 weeks) was started and the patient was addressed to CVID protocol for follow up since then.

**Figure 1:**
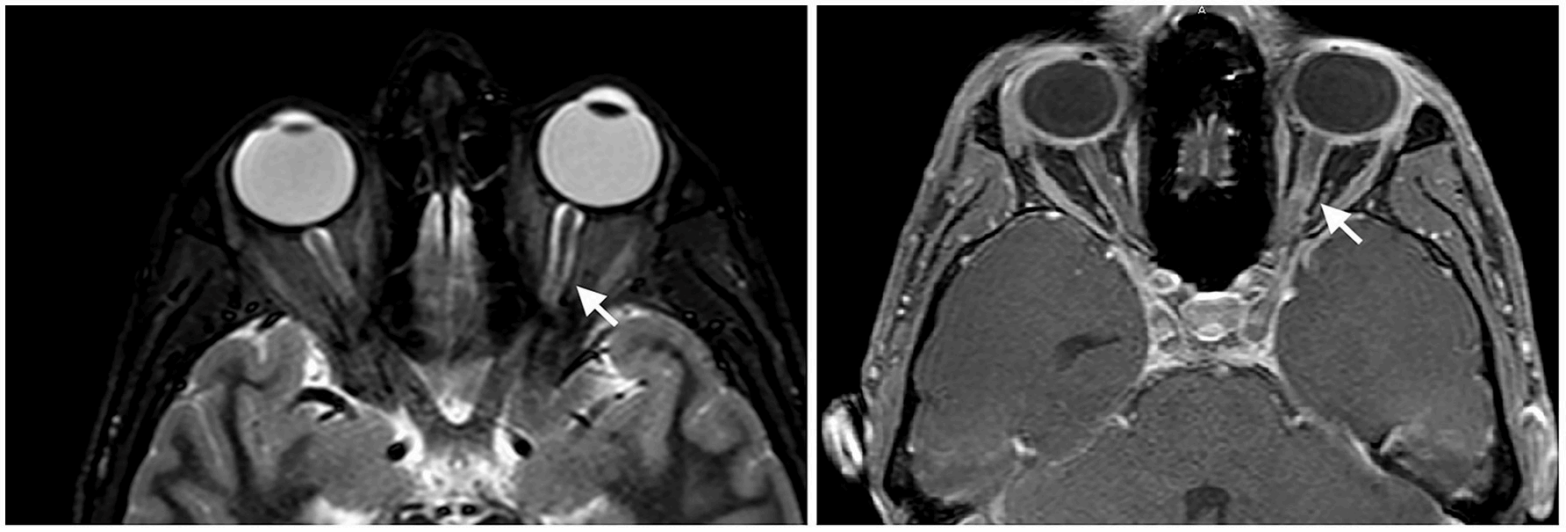
Enlargement and hyperintensity of the optic nerve in fat-suppression MRI techniques (Short-tau inversion recovery or STIR sequence on the left and spectral presaturation with inversion recovery or SPIR sequence on the right). Fat-suppression sequences are useful to identify signal abnormalities of structures surrounded by fatty tissues, such as the optic nerve.

**Figure 2:**
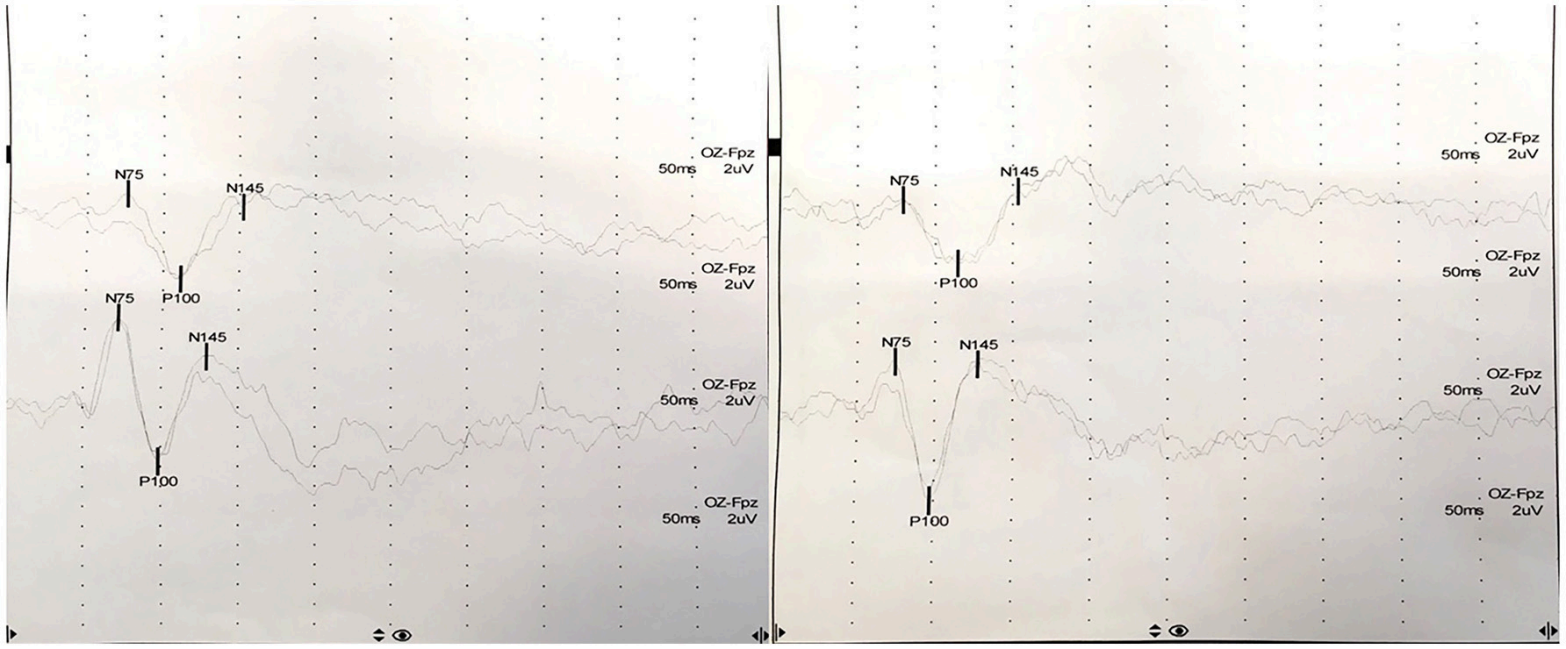
Pattern shift visual evoked potentials showed a marked delay of the P100 component at 15′ (upper) and at 60′ (lower) in the left eye (P100 latency at 15′: 111 ms in the left eye, 98 ms in the right eye; P100 latency at 60′: 114 ms in the left eye, 97 ms in the right eye).

At presentation, the patient reported decreased visual acuity and partial loss of color vision on his left eye. Neurologic examination showed reduced visual acuity, relative paracentral scotoma and red desaturation in the left eye, moderate pain on pressure of the ipsilateral ocular bulb, but no pain on ocular movements. Pupillary light reflex was reduced on the same side, and relative afferent pupillary defect or Marcus-Gunn pupil was present. The right eye was unaffected and the remaining neurological examination was unremarkable. Fundoscopic evaluation revealed a sharp-edged optic disk in absence of stasis signs and retinal lesions. Optical coherent tomography (OCT) showed normal retinal morphology and thickness. Brain and spinal cord Magnetic Resonance Imaging (MRI) demonstrated enlargement and hyperintensity of the left optic nerve in its posterior portion, with intense enhancement after the administration of gadolinium (Figure 1). Pattern shift visual evoked potentials showed a marked delay of the P100 component with an attenuation of the P100 wave amplitude in the left eye (Figure 2). Cerebral spinal fluid (CSF) analysis revealed marked pleocytosis (200 cells/ul) with lymphocyte prevalence, and mildly elevated protein level (56 mg/dl). Oligoclonal bands were absent. PCR for *HIV, HSV 1, HSV2, HHV6, HHV8, EBV, VZV, Parvovirus, Adenovirus, Enterovirus, BKV, Pneumococcus, Streptococcus group B, Cryptococcus, and Toxoplasma* did not detect any infection. CSF cultures were negative. Serum anti-AQP4 and anti-MOG antibodies were absent. Likewise, anti-nuclear antibodies, anti-extractable nuclear antigens antibodies, anti-double strand DNA antibodies, and anti-tireoperoxidase antibodies turned out negative.

Routine blood examination showed mild leukopenia (3,250/ul) and low platelet count (72,000/uL). Serum IgG and IgM levels were, respectively, 764 mg/dL and 8 mg/dL, while serum IgA were undetectable. Circulating lymphocytes were 1463/μL, (CD3+ 835/μL, CD4+ 559/μL, CD8+ 240/μL, CD19+ 536/μL, CD16+56+ 84/μL). Lymphocytes immunophenotyping and maturative analysis showed decreased NK cells (84/uL), failure of maturation of B lymphocytes with markedly reduced switched memory B cells (0.6%), transitional B cells (0.1%) and plasmablasts (0.2%), and further increased CD21 low subpopulation (36.7%). Infection markers resulted within normal range. Serum β2-microglulin was slightly increased compared to baseline (4.09 mg/dL; n.r. 0.8–2.2). Serum PCR for *HIV, HSV 1, HSV2, HHV6, HHV8, EBV, VZV, Parvovirus, Adenovirus, Enterovirus, BKV, Pneumococcus, Streptococcus group B, Cryptococcus*, and *Toxoplasma* were negative. Serum serologies for *Treponema pallidum, Borrelia burgdorferi, Bartonella henselae*, and *Brucella* were negative, as well as QuantiFERON gold test for *Mycobacterium tubercolosis* and serum galactomannan assay for *Aspergillus* detection.

Given the higher susceptibility of CVID patients to lymphoproliferative diseases, cytological and radiological examinations were carried out in order to exclude CNS lymphoma. No malignant cells were detected in the CSF. Total body Positron Emission Tomography (PET)/CT study with 18-FDG did not show any focal increase in glucose metabolism.

As soon as viral infections were excluded, our patient was treated with an empiric course of IV methylprednisolone (1 g/die for 5 days) and a tapering course of prednisone, as early IV steroidal therapy was shown to accelerate clinical resolution in patients with autoimmune ON. IV ceftriaxone and oral Bactrim were added while waiting for microbial cultures results. Given the history of Zoster reactivation, prophylactic therapy with oral Acyclovir was administered to the patient in concomitance with steroidal therapy. The patient reported a rapid clinical improvement following the administration of the first methylprednisolone dose, with complete regression of symptoms in 4 days, and remained asymptomatic at 6-month follow-up.

## Discussion

Although long known, the pathophysiology of CVID has not been completely elucidated yet. Despite few monogenic defects have been identified in a subset of CVID patients, the clinical heterogeneity of this condition suggests that it is a polygenic disorder which encompasses multiple molecular alterations resulting in a common final endpoint of hypogammaglobulinemia and dysregulated immune response (1, 15, 16). Primary B-cell dysfunction and defects in T-cells and antigen-presenting cells can be observed (17). Moreover, B lymphocytes typically fail to differentiate into switched memory B cells and plasma cells, and a reduction in switched memory B cells is invariably observed (15, 18). Notably, switched memory B cells develop in the germinal center in a T-dependent fashion, therefore their reduction could be ascribed both to T and B cells dysfunctions (15, 17). Indeed, several abnormalities in CD4+ and CD8+ T cells proliferation, activation, survival and secretory functions have been found in these patients (15, 17, 18). Moreover, suppressive functions of T-regulatory (Treg) cells are altered (19). These alterations are associated with a chronic immune activation and with high incidence of autoimmunity.

Indeed, although it may seem paradoxical, CVID is characterized by an increased susceptibility to autoimmunity, malignancies and inflammatory conditions (20). Autoimmune cytopenias are diagnosed in almost 25% of CVID patients and can be the presenting disorder (18, 21). Autoimmunity is a manifestation of immune dysregulation, but its precise mechanisms in CVID are still unclear. Expansion of CD21 low B cells and reduction in Treg cells is frequently observed in CVID and is associated with a clinical phenotype characterized by autoimmunity and splenomegaly (22–24). Treg cells are a subset of T cells able to produce cytokines, such as IL-10, IL-35, and TGFβ, which suppress pro-inflammatory responses (25). Treg cells induce the conversion of dendritic cells to a tolerogenic state which hampers IL-2-dependent activation of CD8+ T cells and natural killer cells (25).

Furthermore, a reduction in IL10-producing B-regulatory (Breg) cells compartment has been reported in CVID patients in two studies (26, 27). Breg cells are responsible for negative modulation of immune responses, and their dysregulation or absence may trigger inflammatory or autoimmune diseases (26, 28). The regulatory functions of these B cells are mainly associated with their production of IL-10 and TGF-β, which stimulate the expansion of Treg cells, restore TH1/TH2 balance, and inhibit TH17 cells (26, 28). Moreover, there is evidence that the number and abilities of Breg cells increase in response to inflammation and autoimmunity, thus limiting the spread and severity of these processes (28). Therefore, the reduction of Treg cells and failure in Breg compartment expansion and activation might account for the increased incidence of autoimmune diseases in CVID patients. Other alterations that can be observed include impaired somatic hypermutation in B cells, and defects in dendritic cells functions (29).

Despite antibody production in response to antigens is reduced or even lacking, the generation of autoantibodies might, at the same time, be excessive (15). Thus, testing for autoantibodies levels should be performed and may be a useful tool for diagnosis, whenever there is clinical suspicion, although a negative result does not exclude autoimmunity. Conversely, detection of infectious agents in CVID patients generally requires the use of molecular diagnostic techniques, as antibody response to exogenous antigens is ineffective and the use of immunoglobulin replacement therapy may further challenge the validity of serological findings (1).

Patients with CVID should be monitored periodically in experienced centers. They should receive adequate replacement therapy with immunoglobulins, physiotherapy, and prophylactic antibiotic therapy if necessary, and they should be promptly diagnosed and treated for any CVID complications which could arise during follow up (2).

Optic neuritis (ON) is rarely infectious in nature (30–32). Frequently, optic nerve involvement may occur in concomitance with neuroretinis, characterized by optic disc edema and macular exudates (30). However, given their increased susceptibility to infections, microbial agents must be thoroughly ruled out in CVID patients. Among infectious causes of optic neuropathy, the most commonly isolated agents are *Bartonella henselae, Borrelia burgdorferi, Treponema pallidum, Mycobacterium tuberculosis, Herpesviruses, Arboviruses, Toxoplasma* (14, 30–32). In our patients, a wide range of possible infectious causes of ON have been investigated and excluded.

Isolated ON may be the presenting symptom of MS (33). Patients with no lesions on baseline MRI present a 25% risk of developing clinically definite MS in the long term, comparing to 75% risk of patients with abnormal scans (33). Among patients with normal baseline MRI, further risk factors are female sex, normal optic disc appearance, and typical clinical features (33).

A less common cause of isolated ON is NMOSD. AQP4 antibodies can be detected in approximately 5.8% of patients with acute ON (34). NMOSD is characterized by recurrent attacks of unilateral or bilateral optic neuritis, transverse myelitis, and acute brainstem or area postrema syndromes (12). Overall, AQP4 antibodies are detectable in ~88% of patients with clinical features of NMOSD (35). Detectable serum MOG autoantibodies can be found in a minority of patients with clinical characteristics of NMOSD, and their presence is associated with younger age at onset, male sex and a reduced risk of recurrence comparing to patients with AQP4 seropositivity (36, 37). In our patient, serological analysis for AQP4 and MOG autoantibodies turned out negative.

In addition to that, ON can occur in a variety of systemic inflammatory conditions, such as Systemic Lupus Eritematosus, Sjogren's syndrome, and granulomatosis with polyangiitis (30). In these cases, additional clinical findings and associated autoantibodies may aid in the diagnosis. Furthermore, a great mimicker such as sarcoidosis may present with a typical ON (30). These diseases seemed unlikely in our patient in view of the negative serologic studies and normal PET/CT scans.

Although rare, optic neuropathies may arise in the setting of primary CNS Non-Hodgkin lymphomas (NHL) and paraneoplastic syndromes (38–43). As in CVID there is an increased risk of developing NHL (4, 44, 45), total body PET/CT and CSF analysis were performed, yielding negative results.

Polyclonal lymphocytic infiltration (i.e., granulomas, unexplained hepatomegaly, persistent lymphadenopathy, splenomegaly, lymphocytic enteropathy, lymphoid interstitial pneumonitis) is a potential CVID-related complication (2, 45, 46). Nevertheless, lymphocytic infiltration of the CNS is extremely rare. In a case series, possible radiologic findings were mass lesions, leptomeningeal enhancement, non-specific white matter lesions, and neurohypophysis abnormalities, and most common clinical presentations were seizures, headache, vision loss, cognitive impairment, and focal deficits (46). Most pathologic lesions consisted of granulomatous formations. CSF findings include increased total proteins with or without lymphocytic pleocytosis. Treatment of CNS granulomatous disease is empirical and based on scant case reports or case series (2, 46). Steroids, intravenous immunoglobulins and other immunosuppressive agents have been tried, alone and in combination, mostly leading to recovery in the short term. Our patient displayed a clinical phenotype of systemic polyclonal lymphocytic infiltration, therefore CNS involvement should be held into account, although isolated optic nerve infiltration has never been described and increased uptake in PET scans is expected in case of lymphomatous granulomatosis.

Having ruled out other possible causes of ON, the occurrence of a clinically isolated syndrome (CIS) appears as the most likely diagnosis. As mentioned, the risk of developing MS in the long term is around 25% in patient with CIS and a normal baseline MRI scan (33). The diagnosis of MS requires clinical or radiological evidence of dissemination in time and space, or the presence of oligoclonal bands in the CSF (47), therefore clinical and instrumental follow-up is needed in these cases. Early intravenous methylprednisolone at high doses (1 g/die) was shown to accelerate visual recovery and reduce the risk of conversion to MS in the first 2 years after ON, but no differences in comparison with controls were seen in the long run (48–50). Patients with typical, isolated ON without further MRI lesions are usually monitored, and MRI performed at regular intervals might enable and earlier diagnosis of MS (11). If the disease converts to MS, relapsing in the subsequent 2 years, then disease-modifying drugs can be prescribed (11).

MS is a complex and heterogeneous disease whose pathogenesis has not been completely clarified yet (51). It was originally considered a T cell-mediated disease, but evidence regarding the involvement of additional cell types, including B cells, has been accumulating over the years (52). The pathogenic process appears to begin with the activation of peripheral autoreactive T cells by mimicry or bystander activation, followed by their infiltration into the CNS, where they lead to the activation of additional cells, such as B cells, monocytes, astrocytes, and microglia (52). Several studies suggest that Breg cells might be involved in MS pathogenesis. Indeed, B cells from MS patients were shown to have a significantly diminished capacity to secrete IL-10 in response to stimuli (53–55). However, other studies did not confirm these results (56, 57). These contrasting findings might be due to the use of different stimulation protocols, different definitions of Bregs, and variations in patients cohorts and MS subsets (52). Several studies have revealed that declines in the number and functions of Treg cells contribute to the development of autoimmune diseases (22, 24, 25). A recent work showed that both naïve and memory Treg cells are reduced in in MS patients and that Treg cells mainly express the FoxP3 isoform lacking exon 2 (58). Additionally, they observed increased membrane levels of PD-1, the inhibitory receptor of Treg subsets (58). Taken altogether, these findings seem to suggest a potential role of B and T regulatory cells in the pathogenesis of autoimmune demyelinating CNS disorders, which were previously shown to be involved in the pathogenesis of autoimmune complications of CVID. Whether these cells play a role in prevention of development of autoimmune diseases or only in containing and cessating altered immune responses is not clear. However, they might represent a potential pathogenic link between these two distinct conditions.

## Concluding remarks

To date, there is only one report of ON in a patient with CVID, presenting as bilateral clinically isolated demyelinating syndrome (10). Our case further strengthens this association, thus encouraging clinicians to consider the possibility of acute demyelination in CVID patients with unilateral ophthalmological symptoms. Establishing a connection between these diseases may have a significant impact on clinical practice and patients' well-being, as early steroidal therapy in ON results in accelerated clinical improvement and reduced risk of developing MS in the subsequent 2 years (48–50). However, as CVID patients are prone to infections and hematologic malignancies as well, these conditions need to be carefully excluded on the basis of clinical, laboratory, and instrumental findings.

In conclusion, neurological manifestations in CVID are challenging for both their atypical presentation and for the nature of the disease itself, which can provoke or mimic several diseases (2, 9). Autoimmune diseases, especially cytopenias, occur in 25% of CVID patients, and splenomegaly is also a common finding in such patients. Dysregulation in suppressive function of regulatory T and B cell subsets has been described in association with these clinical findings. Quantitative and qualitative alterations in these cellular populations might represent the link between CNS demyelination and CVID. However, further studies are needed in order to better elucidate these connection. CVID remains a largely obscure pathologic entity, and a better comprehension of its pathogenic mechanisms and clinical manifestations is essential to better understand and treat this severe condition.

## Ethics statement

The case report has been performed in accordance with the ethical standards laid down in the 1964 Declaration of Helsinki and its later amendments. Informed written consent for publication was obtained from the participant prior to the inclusion in the study.

## Author contributions

All the authors took care of patient management and made decisions about patient treatment. EA and IF conceived the idea and collected the clinical data. EA revised all the literature and wrote the manuscript. IF made revisions to the manuscript by adding important intellectual content. MC analyzed immunological data and made revisions to the manuscript by adding important intellectual content. FM, AG, DV, DG, EM, and RB collected the clinical data and contributed to the writing of the manuscript. NB, GF, GC, and SC contributed to the revision of the manuscript, read, and approved the submitted version.

### Conflict of interest statement

The authors declare that the research was conducted in the absence of any commercial or financial relationships that could be construed as a potential conflict of interest.

## Abbreviations

AQP4: Acquaporin 4
CNS: Central Nervous System
CSF: Cerebral spinal fluid
CT: Computed Tomography
CVID: Common Variable Immunodeficiency
Ig: Immunoglobulin
MOG: Myelin oligodendrocyte glycoprotein
MRI: Magnetic Resonance Imaging
MS: Multiple Sclerosis
NHL: Non-Hodgkin lymphoma
NMOSD: Neuromyelitis Optica Spectrum Disorder
OCT: Optical Coherence Tomography
ON: Optic Neuritis
PET: Positron Emission Tomography.
